# Evaluating the construct validity and test-retest reliability of the Orthotic Patient-Reported Outcomes–Mobility (OPRO-M) short forms in lower limb orthosis users

**DOI:** 10.1371/journal.pone.0330334

**Published:** 2025-08-19

**Authors:** Geoffrey S. Balkman, Phillip M. Stevens, Eric L. Weber, Alyssa M. Bamer, Rana Salem, Bretta L. Fylstra, Sara J. Morgan, Brian J. Hafner

**Affiliations:** 1 Department of Rehabilitation Medicine, University of Washington, Seattle, Washington, United States of America; 2 Hanger Institute for Clinical Research and Education, Austin, Texas, United States of America; 3 Department of Physical Medicine and Rehabilitation, University of Utah, Salt Lake City, Utah, United States of America; 4 Gillette Children’s Specialty Healthcare, St. Paul, Minnesota, United States of America; 5 University of Minnesota, Minneapolis, Minnesota, United States of America; Iran University of Medical Sciences, IRAN, ISLAMIC REPUBLIC OF

## Abstract

Lower limb orthoses (LLOs) are often prescribed to facilitate mobility in individuals with functional impairments. The Orthotic Patient-Reported Outcomes – Mobility (OPRO-M) is a self-report instrument developed recently to measure LLO users’ perceived mobility with an orthosis. An observational, prospective, psychometric validation study was conducted to evaluate the construct validity and test-retest reliability of the OPRO-M 12- and 20-item short forms. LLO users were recruited from orthotic clinics across the United States. Participants were administered four self-report instruments (OPRO-M, Orthotic and Prosthetic Users Survey – Lower Extremity Functional Status, Lower Extremity Functional Scale, and Patient-Reported Outcomes Measurement Information System – Physical Function) and three performance-based instruments (10-meter Walk Test, Timed Up and Go Test, and Two-Minute Walk Test) during an in-person assessment. Self-report instruments were re-administered via an online survey sent to participants 7 days later. Convergent validity was assessed by comparing OPRO-M scores to those from co-administered self-report and performance-based instruments. Known groups validity was evaluated by comparing scores from patients grouped by clinician-assigned mobility level. Test-retest reliability was assessed by comparing scores from the in-person and follow-up assessments. Standard error of measurement (SEM) and smallest detectable change (SDC) were derived from test-retest reliability coefficients. A total of 104 LLO users (51% male, mean age = 53 years) completed both assessments. OPRO-M short form scores correlated strongly with those from self-report (ρ = 0.84–0.91) and performance-based (|ρ| = 0.73–0.83) instruments. OPRO-M short form scores also effectively differentiated all mobility groups except household and limited community ambulators. The OPRO-M short forms showed excellent test-retest reliability (ICC = 0.93–0.94) and low measurement error (SEM = 2.4–2.6, SDC = 5.5–6.0). These results provide sound evidence of the OPRO-M short forms’ validity and reliability when used to measure mobility in LLO users. These instruments are promising, population-specific alternatives to generic surveys with psychometric performance comparable to or better than established self-report instruments.

## Introduction

Lower limb orthoses (LLOs) are often prescribed to individuals with functional impairments to address their mobility limitations [1]. These externally-applied braces serve as key rehabilitative interventions for people with a wide variety of health conditions [2]. The clinicians responsible for providing lower limb orthotic care (i.e., orthotists and physical therapists) are therefore often motivated to measure their patients’ mobility in order to evaluate the effectiveness of the LLOs patients receive. Self-report instruments (i.e., surveys) are a type of standardized outcome measure well suited to assessing LLO users’ mobility as they solicit information about how orthoses affect a patient’s functional abilities at home or in their community. Self-report instruments are also advantageous because they can measure a LLO user’s perceived ability to navigate situations or environments that are difficult to recreate in a clinical setting (e.g., going for an all-day hike). They are often appealing to clinicians because they are inexpensive, easy to administer, and require little training to use [3].

There are two basic types of self-report instruments used in clinical practice, generic and population-specific [4]. Generic self-report instruments are intended to measure health outcomes germane to people with a range of health conditions. Population-specific instruments are designed to measure aspects of health relevant to people with specific health characteristics or shared experiences (e.g., LLO users). Population-specific instruments are limited in that they are generally only applicable for the groups for which they are designed, but they often have greater face validity and credibility [5]. Since they are designed to target issues of importance to specific patients, they are also considered to be more sensitive to changes in the outcome being measured [6]. The Orthotic Patient-Reported Outcomes – Mobility (OPRO-M) is a population-specific instrument designed to assess LLO users’ perceived mobility with their orthosis and any other assistive devices they might use [7,8]. OPRO-M was developed using rigorous methods [9,10] that involved collecting insights from LLO users and obtaining feedback from clinicians [8]. The resulting instrument includes items that describe situations commonly encountered by LLO users (e.g., walking on uneven grass, stepping over an extension cord). The OPRO-M instrument is therefore intended to measure aspects of mobility that are most relevant to LLO users and their care providers.

Initial evidence of OPRO-M’s reliability and validity was established during its development [8]. Reliability (i.e., precision) of OPRO-M was evaluated using the test information curves. The developers determined that the OPRO-M item bank measured with high reliability (i.e., < 0.90) to nearly three standard deviations above and below the mean. Convergent and divergent construct validity of the OPRO-M item bank was evaluated by examining correlations between the OPRO-M item bank scores and scores produced by self-report instruments designed to measure similar and dissimilar constructs, respectively [8]. Known groups construct validity of the OPRO-M item bank was evaluated by examining differences in participants grouped by self-reported characteristics such as fall history and assistive device use. The developers also produced two short forms from the OPRO-M item bank, recommending that these forms be used in clinical care and research. Items in the OPRO-M short forms were selected based on statistical and clinical criteria, balancing important clinical content with high reliability, low reading level, and overall length of the short form [8].

While initial evidence of reliability and validity of the OPRO-M item bank was well established in the development study [8], further research is needed evaluate the psychometric properties of the OPRO-M short forms, the forms of the instrument most likely to be administered by clinicians and researchers. The purpose of this study was therefore to evaluate the construct validity and test-retest reliability of the OPRO-M short forms in an independent clinical sample of LLO users. We hypothesized that OPRO-M short form scores would correlate strongly (|ρ| ≥ 0.7) with scores from other self-report instruments that measure related constructs. We anticipated that OPRO-M short form scores would correlate moderately (0.3 ≤ |ρ| ≤ 0.7) with scores from performance-based instrument scores that measure distinct aspects of mobility. We also hypothesized that the OPRO-M short forms would demonstrate excellent reliability (ICC ≥ 0.90), supporting their use for individual-level clinical decision-making. Results of this study will provide valuable psychometric evidence for OPRO-M short forms when applied to LLO users in routine clinical practice.

## Materials and methods

### Study design

An observational, prospective, psychometric validation study was conducted at multiple sites between September 2022 and October 2023 to evaluate the construct validity and test-retest reliability of the OPRO-M short forms. Convergent construct validity was assessed by comparing OPRO-M short form scores to scores obtained from other self-report and performance-based instruments. Known groups construct validity was assessed by comparing OPRO-M short form scores across LLO users grouped according to clinician-rated mobility. Test-retest reliability was evaluated by comparing scores from repeated administrations of OPRO-M short forms over a short period of functional stability. Indices of measurement error (i.e., standard error or measurement [SEM] and smallest detectable change [SDC] were derived from test-retest correlation coefficients. Results of this study are presented in accordance with the COnsensus-based Standards for the selection of health status Measurement INstruments (COSMIN) reporting guideline for studies on measurement properties of patient‑reported outcome measures [11]. All study procedures were approved by the University of Washington Human Subjects Division. All participants were informed of study procedures and provided written informed consent prior to beginning the study.

### Participants

Convenience sampling was used to recruit LLO users from orthotic clinics across the United States. Individuals who had previously received a LLO from participating clinics were contacted by phone to gauge interest and screen for eligibility. Individuals were eligible to participate if they were 18 years of age or older, were able to read and write in English, were prescribed an orthosis that extended proximally from the foot to a level above the ankle for one or both legs, had used orthosis(es) for at least one month, were currently using their orthosis(es) most days of the week, were able to stand or walk without help from another person, and had access to an electronic device with internet access. Individuals with major upper or lower limb amputations or those who were using a temporary LLOs for acute injuries (e.g., walker boot, figure-of-eight ankle wrap) were not eligible to participate in the study.

Sample size was estimated using α = 0.05, β = 0.80, ρ_0_ = 0.00, ρ_1_ = 0.30, and two tails in a bivariate normal model in G*Power 3.1.9.2 (Keil, Germany). An expected minimum correlation of ρ_1_ = 0.30 between self-report and performance-based instrument scores was chosen based upon previously-reported correlations between timed walking tests and the Lower Extremity Functional Scale (LEFS) (|ρ| = 0.32–0.69) [12–15] and the Patient-Reported Outcomes Measurement Information System – Physical Function (PROMIS-PF) (|ρ| = 0.43–0.58) [16–18] in people with orthopedic, cardiovascular, or neurological injuries. The sample size of 84 suggested by the power analysis was increased to 100 to meet the minimum sample recommended by the COnsensus-based Standards for the selection of health Measurement INstruments (COSMIN) for a “very good” study design quality rating [19].

## Measures

### Self-report instruments

The OPRO-M item bank is a self-report measure of mobility designed for use with adults who use LLOs [8]. OPRO-M includes 39 items calibrated to an item response theory (IRT) statistical model. Each item begins with, “Are you currently able to…,” followed by a specific activity that requires use of lower limbs, ranging from basic ambulation (e.g., walking a short distance at home) to more difficult activities (e.g., going for an all-day hike). The five response options reflect the degree of difficulty with which respondents report they can carry out each activity, ranging from “Unable to do” to “Without any difficulty.” OPRO-M can be administered as either a 12- or 20-item short form. The 20-item short form was administered in this study. However, as all items in the 12-item short form are included in the 20-item short form, the reliability and validity of both forms can be tested using the collected data. OPRO-M summary scores ranging from 12 to 48 (i.e., 12-item short form) or from 20 to 80 (i.e., 20-item short form) are converted to standardized T-scores using lookup tables specific to each form. A T-score of 50 (SD = 10) represents the mean mobility reported by the OPRO-M development sample, which included a broad range of individuals (n = 1036) who use orthoses [8]. Psychometric testing in the development sample showed that OPRO-M has preliminary evidence of convergent and known groups construct validity in individuals who use ankle-foot orthoses (AFOs) and knee-ankle-foot orthoses (KAFOs) [8].

Three self-report instruments that have been previously used to evaluate mobility or physical functioning in LLO users were co-administered to evaluate OPRO-M’s convergent construct validity. These included the Orthotic and Prosthetic Users’ Survey – Lower Extremity Functional Status (OPUS-LEFS) [20], a population-specific fixed-length survey instrument; the LEFS [21], a population-generic fixed-length survey instrument; and the PROMIS-PF [22], a population-generic short from derived from an IRT-calibrated item bank.

The OPUS-LEFS includes 20 items for evaluating the physical functioning of individuals who use LLOs or lower limb prostheses. Respondents are provided with the context, “How easy, or difficult, is it for you to…” Each item describes an activity (e.g., “get up from the floor”) and includes five response options ranging from “Very easy” to “Cannot do this activity.” Summary scores ranging from 0 to 80 are converted to a standardized Rasch measure (ranging from 0 to 100) using a lookup table. Higher Rasch measure indicate higher levels of physical function. OPUS-LEFS has been shown to have evidence of internal consistency when tested with a mixed sample of orthosis and prosthesis users [20]. The OPUS-LEFS has also exhibited evidence of content validity, construct validity, and test-retest reliability in AFO users [23].

The LEFS also consists of 20 items, each with the context, “Today, do you or would you have any difficulty at all with…” Each item describes an activity (e.g., “walking between rooms”) with five response options ranging from, “No difficulty” to “Extreme difficulty or unable to perform activity.” Summary scores range from 0 to 80, and higher scores indicate higher levels of physical function [21]. LEFS has been shown to have evidence of validity and reliability when administered to people with orthopedic disorders [24] and those affected by stroke [14].

The PROMIS-PF is an IRT-calibrated item bank developed to measure perceived physical functioning in a broad range of individuals. PROMIS-PF [22]. PROMIS-PF instruments are scored on a T-score metric centered (mean = 50) on a large sample representative of the U.S. general population. Each item begins with the context of, “Are you able to…” or “Does your health now limit you in…” and includes five response categories ranging from “Without any difficulty” to “Unable to do” or “Not at all” to “Cannot do,” respectively. Examples of activities include “stand for one hour” and “go up and down stairs at a normal pace.” The PROMIS-PF 20- item short form version 2.0 was administered as it is comparable in length to the other self-report instruments included in this study. Scores for the 10-item short form were also derived as all items are included in the 20-item form. PROMIS-PF summary scores ranging from 20 to 99 (20-item short form) or from 10 to 50 (10-item short form) are converted to T-scores using standardized lookup tables. Higher scores indicate better physical function. PROMIS-PF has demonstrated evidence of content validity, construct validity, and test-retest reliability in AFO users [23]. It has also been shown to have strong psychometric evidence when tested with people diagnosed with lower extremity health conditions [25], spine disorders [26], ischemic stroke [27], and multiple sclerosis [28].

Questions about demographics (e.g., age, sex, race and ethnicity, military status), health conditions, and orthosis and assistive device use (e.g., history of use, typical weekly and daily use) were also included with the self-report instruments used to characterize the study sample.

### Performance-based instruments

Three performance-based instruments that are commonly used in orthotic clinical care and research were also included in the study to further evaluate OPRO-M’s convergent construct validity. These included the Timed Up and Go Test (TUG) [29], the 10-meter Walk Test (10mWT) [30], and the Two-Minute Walk Test (2MWT) [31]. Standardized protocols were developed for each performance-based instrument to improve the consistency of administration across clinical sites (S1 File).

The TUG is a test of functional mobility that was originally designed to assess fall risk in frail older adults [29]. The TUG requires the person to stand up from a chair with arms, walk at a comfortable speed for 3 meters, turn around, and return to sit in the chair. Performance is timed and faster TUG times are indicative of better mobility. In this study, an average time was calculated from two trials. The TUG has been shown to have evidence of inter- and intra-rater reliability in people with a variety of health conditions, including spinal cord injury [32], stroke [33], and multiple sclerosis [34].

The 10mWT is a test of self-selected walking speed first used to measure recovery after stroke [30]. The 10mWT requires the person to walk at a comfortable speed over a distance of 10 meters. Faster 10mWT speeds are indicative of better performance. In the current study, participants started walking 2 meters behind the 0-meter mark and stopped walking 2 meters beyond the 10-meter mark, consistent with the protocol developed by Cheng et al. for evaluating walking speed in post-stroke patients [35]. The average speed from two trials was calculated. The 10mWT has evidence of test-retest reliability when administered to people with traumatic brain injury [36] and spinal cord injury [37].

The 2MWT is test of walking endurance [31], and is a shorter version of the 12-minute walking test [38]. The 2MWT requires that the person walk as far as possible over two minutes. Longer walking distances indicate better endurance. In this study, participants performed a single 2MWT trial by walking around two cones placed 10 meters apart. The 2MWT has evidence of test-retest reliability in people with neurological impairment [39], post-polio [40], and stroke [41].

### Clinician-reported information

The orthotists who conducted the in-person assessment rated each participant’s mobility level based on their current presentation. A *home ambulator* was defined as one who may not be able to enter or leave the home independently; has difficulty with curbs, stairs, and uneven terrain; and may need assistance or use of a wheelchair to perform activities around the home. A *limited community ambulator* was defined as one who can enter and leave the home independently; can ascend and descend curbs independently; can manage stairs to some degree; and may need assistance or use of a wheelchair to perform more advanced community activities. An *unlimited community ambulator* was defined as one who can ascend and descend stairs independently; can navigate crowds and walk over uneven terrain; can engage in more advanced community activities without assistance or use of a wheelchair. Lastly, an *active adult or athlete* was defined as one who can engage in sports and recreational activities. The orthotist also provided information about the participant’s orthosis (e.g., orthosis type, laterality) to assist with characterizing the study sample.

### Procedures

Clinicians at each participating site were trained to carry out in-person assessments prior to enrolling participants. Each clinic received a kit with standardized testing materials, including a tablet computer, measuring tape, stopwatch, cones, tape, and setup instructions for the performance-based instruments. An investigator used video conferencing to confirm accurate set up of the testing area and observe each clinician practicing administration of the performance-based instruments.

At the in-person assessment, the clinician or designated clinical staff first administered the self-report instruments on a tablet computer (iPad, Apple Inc, Cupertino, CA). The trained clinicians then administered the three performance-based instruments. Participants were emailed a link to an online survey containing the self-report instruments 7 days after the in-person assessment. Participants were expected to remain clinically stable during this period. Participants were sent up to 3 reminders (2 by email, 1 by phone) to complete the survey. The surveys and clinician data collection forms were programmed in and administered using a secure REDCap database [42] hosted the University of Washington.

### Data analysis

Two investigators reviewed each participant’s survey responses for inconsistencies or major changes in specific health outcomes (e.g., pain interference) to verify clinical stability between the in-person and follow-up assessments. All statistical analyses were conducted with SPSS Statistics v.29 (IBM, Armonk, NY). A threshold of significance was set *apriori* at α = 0.05.

### Validity

Convergent construct validity was examined by calculating Spearman rank correlations between OPRO-M short forms and each of the comparison self-report and performance-based instruments. Correlation coefficients were evaluated using established thresholds to identify evidence of strong, moderate, or weak correlation [43]. Correlations in the expected direction, and with an absolute magnitude above 0.7 (i.e., between OPRO-Ms short form and other self-report instruments) or between 0.3 and 0.7 (i.e., between OPRO-M short forms and performance-based instruments), provided evidence of convergent construct validity. Known groups construct validity was evaluated by comparing OPRO-M short form scores across participants grouped by clinician-rated mobility. A one-way analysis of variance (ANOVA) was used to evaluate differences in OPRO-M scores, and Tukey post-hoc pairwise comparisons were performed to identify subgroups with significant differences.

### Reliability and measurement error

Per established recommendations for reliability testing of self-report instruments [44], test-retest reliability was examined by calculating the intraclass correlation coefficient (ICC; 2-way mixed-effects, absolute agreement). Computed ICCs were compared relative to the ≥ 0.90 recommended threshold for individual-level applications (e.g., patient decision-making) [45]. The standard error of measurement (SEM) was calculated to estimate the amount of error in cross-sectional applications, and the smallest detectable change (SDC) was calculated to estimate the amount of error in longitudinal measurements. The SEM was calculated as SD_pooled_ x √(1 – ICC) [46]. The SDC was calculated as 1.65 x √2 x SEM and 1.96 x √2 x SEM for the 90% and 95% confidence intervals, respectively.

## Results

A total of 121 LLO users from 19 orthotic clinics completed the in-person (i.e., test) assessment and 116 participants completed the follow-up (i.e., retest) survey. Data from 12 participants were excluded from the final dataset due to reported changes in health or mobility between the in-person and follow-up assessments. The final dataset therefore included 104 participants with various health conditions (Table 1). The mean age of the study sample was 53 years, 51% were male, and 75% reported being non-Hispanic and white. Most participants used an AFO for one or both legs (n = 94), and the health conditions reported most frequently were stroke and spinal cord injury. The sample included individuals from each mobility category, including household ambulator (12%), limited community ambulator (22%), unlimited community ambulators (50%), and active adult or athlete (16%). The time between the test and retest surveys ranged from 7 to 16 days (mean = 9 days, SD = 2 days).

**Table 1:** Demographic, health, and orthosis characteristics of study participants (n = 104 LLO users).

*Characteristic*	*n*	*%*	*Characteristic*	*n*	*%*
*Sex*			*Health condition*		
Female	53	51	Stroke	15	14
Male	50	48	Spinal cord injury	10	10
Other / not specified	1	1	Peripheral neuropathy/foot drop	9	9
*Ethnicity*			Charcot-Marie-Tooth disease	8	8
Hispanic or Latino	9	9	Traumatic orthopedic leg injury	8	8
Not Hispanic or Latino	91	88	Nontraumatic orthopedic leg condition	7	7
Prefer not to answer	4	4	Post-polio syndrome	5	5
*Race*			Spina bifida	5	5
American Indian or Alaskan Native	1	1	Cerebral palsy	4	4
Asian	5	5	Traumatic brain injury	4	4
Black or African American	6	6	Muscular dystrophy	4	4
Native Hawaiian or Other Pacific Islander	1	1	Multiple sclerosis	3	3
White	82	79	Neuromuscular disease	3	3
More than one race	2	2	Surgery-related nerve injury	3	3
Prefer to self-describe^*^	2	2	Nontraumatic spinal condition	2	2
Prefer not to answer	5	5	Congenital limb difference	1	1
*Employment status*			Other condition^**^	7	7
Employed (or self-employed)	36	35	More than one condition	6	6
Homemaker	6	6	*Orthosis type*		
Student	25	24	Unilateral AFO	64	62
Unemployed	28	27	Bilateral AFO	28	27
Retired	3	3	Unilateral KAFO	10	10
On disability	4	4	Bilateral KAFO	0	0
Prefer not to answer	2	2	AFO and KAFO	2	2

Percentages may not sum to 100% due to rounding. AFO: ankle-foot orthosis, KAFO: knee-ankle-foot orthosis

* Includes Caribbean and Pakistani

** Includes diabetes, vascular disease, complex regional pain syndrome, heart failure, Dandy-Walker syndrome, necrosis, and pressure wound

### Validity

**Fig 1:**
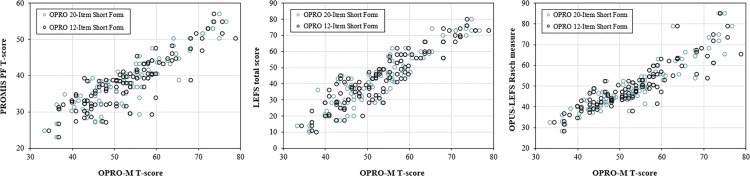
Correlations between OPRO-M short form T-scores and scores from comparison self-report instruments. OPRO-M 20- and 12-item Short Form T-scores were strongly correlated with scores on PROMIS-PF 20-item Short Form (ρ = 0.85 and ρ = 0.84, respectively), LEFS (both ρ = 0.89), and OPUS-LEFS (ρ = 0.91 and ρ = 0.90). Correlations among the comparison self-report instrument scores are located in S1 Fig.

**Fig 2:**
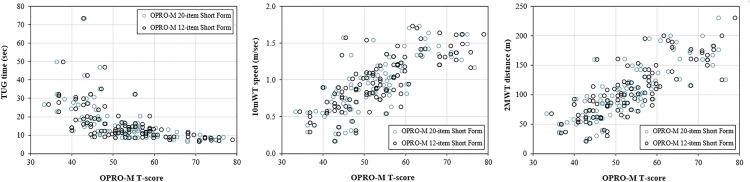
Correlations between OPRO-M short form T-scores and scores from performance-based instruments. OPRO-M 20- and 12-item Short Form T-scores were strongly correlated with TUG times (ρ = −0.74 and −0.73, respectively), 10mWT speed (ρ = 0.77 and 0.75, respectively), and 2MWT distances (ρ = 0.83 and 0.81, respectively). Correlations between comparison self-report instrument scores and performance-based instrument scores are located in S2 Fig.

Scores from the OPRO-M 12- and 20-item short forms were strongly correlated with scores from the PROMIS-PF 20-item short form (ρ = 0.85 and ρ = 0.84, respectively); the LEFS (both ρ = 0.89), and OPUS-LEFS (ρ = 0.91 and ρ = 0.90; Fig 1). OPRO-M short form scores were also strongly correlated with each of the performance-based instruments, including TUG time (ρ = −0.74 and ρ = −0.73), 10mWT speed (ρ = 0.77 and ρ = 0.75), and 2MWT distance (ρ = 0.83 and ρ = 0.81; Fig 2).

One-way ANOVA testing identified statistically significant differences in mean OPRO-M 20- and 12-item short forms between at least two mobility groups (F(2, 2) = [23.3], p **<* *0.001 for both). Post-hoc tests identified significant differences in OPRO-M scores between 5 of 6 mobility group comparisons (Table 2). None of the self-report instruments, including OPRO-M short forms, were able detect significant differences between mobility groups 1 (household ambulators) and 2 (limited community ambulators). Evidence of known groups construct validity from all other instruments is included in Table 2.

**Table 2:** OPRO-M short forms and LEFS detected differences between five of six mobility group comparisons. PROMIS-PF short forms and OPUS-LEFS detected differences between four of six mobility group comparisons.

Self-report instrument	Household ambulator (n = 12)	Limited community ambulator (n = 23)	Unlimited community ambulator (n = 52)	Active adult or athlete (n = 17)	Anova F test (p value)	Differences between mobility groups (p < 0.05)
Mean (SD) Range 95% CI [LB, UB]	Mean (SD) Range 95% CI [LB, UB]	Mean (SD) Range 95% CI [LB, UB]	Mean (SD) Range 95% CI [LB, UB]
OPRO-M 12 (T-score, 0–100)	43.3 (5.6) 34.4 - 55.4 [39.7, 46.8]	47.2 (6.9) 36.8 - 59.8 [44.2, 50.1]	54.6 (8.7) 36.8 - 73.8 [52.1, 57.0]	64.7 (8.4) 50.5 - 78.9 [60.4, 69.0]	23.3 (p < 0.0001)	1–3 1–4 2–3 2–4 3–4
OPRO-M 20 (T-score, 0–100)	42.7 (6.0) 33.3 - 55.5 [38.9, 46.5]	47.3 (6.3) 36.9 - 59.1 [44.6, 50.0]	54.5 (8.7) 36.3 - 74.9 [52.1, 57.0]	64.0 (7.3) 52.2 - 76.7 [52.2, 76.7]	23.3 (p < 0.0001)	1–3 1–4 2–3 2–4 3–4
OPUS-LEFS (Rasch measure, 0–100)	40.1 (6.3) 28.0 - 50.6 [36.1, 44.1]	44.8 (6.7) 31.5 - 59.8 [42.0, 47.7]	50.5 (10.6) 33.1 - 85.0 [47.5, 53.4]	62.3 (10.2) 45.3 - 85.0 [57.1, 67.5]	16.6 (p < 0.0001)	1–3 1–4 2–4 3–4
LEFS (Sum score, 0–80)	29.6 (14.3) 10 - 57 [20.5, 38.6]	34.6 (12.0) 16 - 58 [29.4, 39.8]	44.5 (14.8) 11 - 80 [40.4, 48.7]	60.9 (12.3) 33 - 76 [54.6, 67.3]	16.5 (p < 0.0001)	1–3 1–4 2–3 2–4 3–4
PROMIS PF 10 (T-score, 0–100)	31.2 (4.6) 25.0 - 41.7 [28.3, 34.2]	35.2 (5.0) 26.9 - 42.6 [33.1, 37.2]	39.3 (6.9) 26.0 - 55.8 [37.4, 41.2]	46.1 (7.2) 32.5 - 61.9 [42.4, 49.8]	15.7 (p < 0.0001)	1–3 1–4 2–4 3–4
PROMIS PF 20 (T-score, 0–100)	31.2 (4.9) 23.1 - 41.1 [28.1, 34.4]	34.9 (4.9) 27.1 - 44.2 [33.0, 36.9]	39.3 (6.7) 26.0 - 57.0 [37.5, 41.2]	45.5 (6.4) 31.6 - 54.9 [42.2, 48.8]	16.4 (p < 0.0001)	1–3 1–4 2–4 3–4
TUG (sec)	36.2 (15.4) 16.8 - 73.4 [26.4, 46.0]	19.7 (7.0) 10.2 - 32.3 [16.7, 22.7]	12.5 (3.8) 7.2 - 23.7 [11.5, 13.6]	9.4 (2.2) 6.5–14.7 [8.2, 10.5]	48.9 (p < 0.0001)	1–2 1–3 1–4 2–3 2–4
10mWT (m/sec)	0.5 (0.2) 0.2 - 0.9 [0.3, 0.6]	0.7 (0.2) 0.3 - 1.2 [0.6, 0.8]	1.0 (0.3) 0.6–1.7 [1.0, 1.1]	1.4 (1.1) 1.1–1.7 [1.3, 1.5]	47.6 (p < 0.0001)	1–3 1–4 2–3 2–4 3–4
2MWT (m)	48.8 (18.3) 21.4 - 73.6 [37.2, 60.4]	71.3 (19.7) 30.4 - 109.7 [62.8, 79.8]	114.8 (34.0) 50.0–194.9 [105.4, 124.3]	161.8 (33.6) 119.0–230.0 [144.5, 179.1]	46.2 (p < 0.0001)	1–3 1–4 2–3 2–4 3–4

OPRO-M: Orthotic Patient-Reported Outcomes – Mobility, OPUS-LEFS: Orthotic and Prosthetic Users’ Survey – Lower Extremity Functional Status, LEFS: Lower Extremity Functional Scale, PROMIS PF: Patient-Reported Outcome Measure Information System – Physical Function, SD: standard deviation

### Reliability and measurement error

OPRO-M 12- and 20-item short forms exceeded the 0.90 intraclass correlation coefficient (ICC) threshold for individual-level applications (i.e., 0.93 and 0.94, respectively). The smallest detectable change (with 90% confidence interval) values were 6.0 points for the OPRO-M 12-item short form and 5.5 for the 20-item short form. ICCs also exceeded 0.90 for each of the other self-report instruments, including OPUS-LEFS (0.95), LEFS (0.93), PROMIS PF 10-item short form (0.93) and PROMIS PF 20-item short form (0.94; Table 3).

**Table 3:** Test-retest reliability coefficients and smallest detectable change values for self-report surveys administered to lower limb orthosis users (n = 104). Retest surveys were completed 7 to 14 days after the test survey. All self-report instruments exceeded the 0.90 ICC threshold with a 90% confidence interval. OPRO-M 20- and 12-item short forms, OPUS-LEFS, and PROMIS PF 20-item short form exceeded the 0.90 ICC threshold with a 95% confidence interval.

Self-report instrument	Test		Retest		Absolute difference		ICC (3,1)			SEM	SDC	
Mean	SD	Mean	SD	Mean	SD	ICC	95% CI LB	95% CI UB	SEM	SDC 90	SDC 95
OPRO-M 12	53.3	10.2	52.6	9.6	0.6	0.6	0.93	0.90	0.95	2.6	6.0	7.1
OPRO-M 20	53.1	10.0	52.2	9.7	0.8	0.3	0.94	0.91	0.96	2.4	5.5	6.5
OPUS-LEFS	50.0	11.3	49.0	11.3	1.0	0.1	0.95	0.92	0.96	2.6	6.1	7.3
LEFS	43.3	16.6	41.7	17.1	1.7	0.6	0.93	0.89	0.94	4.6	10.6	12.7
PROMIS PF 10	38.6	7.6	37.6	7.6	1.0	<0.1	0.93	0.89	0.96	2.0	4.5	5.4
PROMIS PF 20	38.4	7.3	37.5	7.3	0.9	<0.1	0.94	0.90	0.96	1.8	4.1	4.9

OPRO-M: Orthotic Patient-Reported Outcomes – Mobility, OPUS-LEFS: Orthotic and Prosthetic Users’ Survey – Lower Extremity Functional Status, LEFS: Lower Extremity Functional Scale, PROMIS PF: Patient-Reported Outcome Measure Information System – Physical Function, SD: standard deviation, ICC: intraclass correlation coefficient, CI: confidence interval, LB: lower bound, UB: upper bound, SEM: standard error of measurement, SDC 90: smallest detectable change with a 90% confidence interval, SDC 95: smallest detectable change with a 95% confidence interval

## Discussion

Results of this study provide strong evidence supporting the validity and reliability of the OPRO-M short forms for measuring mobility in LLO users. As hypothesized, high correlations (ρ ≥ 0.85) were observed between OPRO-M short forms and other self-report instruments measuring similar constructs. The correlations between OPRO-M short form scores and performance-based instruments were somewhat stronger (|ρ| ≥ 0.73) than initially hypothesized, suggesting that these instruments may capture aspects of mobility that align well with these standardized performance-based instruments. Furthermore, test-retest reliability analyses yielded ICCs exceeding 0.90, confirming OPRO-M’s excellent measurement stability over time. Reliability analyses also produced estimates of measurement error that can help clinicians and researchers determine whether changes in mobility due to time, health, or intervention have occurred.

The convergent construct validity of the OPRO-M short forms was strongly supported by its high correlations with established self-report measures of physical function and mobility. The strong relationship between OPRO-M and PROMIS-PF short form scores is particularly noteworthy, as PROMIS instruments undergo rigorous development and validation [9,10]. Strong correlations with the LEFS and OPUS-LEFS also indicate that the OPRO-M short forms effectively measure the mobility construct that these instruments also target. Strong correlations, ranging from 0.87 to 0.92, were also found between OPRO-M item bank scores and those from the PROMIS-PF, LEFS, and OPUS-LEFS survey instruments [8], suggesting that the short forms studied in the current study measure mobility in a manner similar to the full OPRO-M item bank. Correlations from 0.73 to 0.83 between the OPRO-M short forms and the performance-based instruments in the current study further support OPRO-M’s construct validity, indicating that patients’ perceived mobility corresponds well with their demonstrated functional performance. However, a prior study by Bean et al. also suggested that self-report and performance-based instruments likely assess different aspects of physical function [47]. Thus, the clinical information solicited with the OPRO-M is likely still complementary to that obtained from performance-based instruments like the TUG, 10mWT, and 2MWT despite the slightly stronger-than-expected correlations between OPRO-M and the performance-based instruments.

The known groups construct validity analysis revealed that OPRO-M short forms could generally differentiate between LLO users grouped by clinician-rated mobility. In a previous study, scores from the OPRO-M item bank well differentiated participants grouped by characteristics such as level of assistive device use and type of paresis [8]. It was slightly less effective at distinguishing groups based on other characteristics, including type of LLO, number of comorbidities, and number of falls in the past year. Scores from the OPRO-M short forms in the current study were able to differentiate between 5 of 6 comparison groups, but were unable to detect significant differences between household and limited community ambulators. This inability to distinguish between these lower-mobility groups was common to all of the self-report instruments included in the current study, suggesting a potential limitation of these measures when used with individuals with limited mobility. It is also possible that there was overlap in the operational definition of these mobility groups or the small sample of individuals classified as household ambulators in the current study (n = 12) made it difficult to differentiate them from the larger group (n = 23) of limited community ambulators. Interestingly, the TUG was able to differentiate between these lower mobility groups. However, it was unable to differentiate between the two highest mobility groups. A meta-analysis by Schoene et al. similarly found that the TUG was better at discriminating older adults with lower levels of mobility, and less useful for those at higher levels of mobility [48]. This finding reinforces the complementary nature of self-report and performance-based instruments,[3] especially when evaluating patients with a range of mobility limitations.

Test-retest reliability analysis demonstrated that OPRO-M short forms have excellent measurement stability, with ICC values of 0.93 or higher. This high level of reliability exceeds the recommended threshold (≥0.90) for individual-level applications such as patient decision-making [45], supporting use of OPRO-M short forms in clinical settings for individual patient assessment and monitoring. The other self-report survey instruments included in the current study also showed high reliability, suggesting they too have excellent measurement stability. The test-retest reliability of the OPUS-LEFS was also reported to be above 0.90 (i.e., ICC = 0.95) in a recent study specific to AFO users [23]. The reliability of the PROMIS-PF was slightly lower (i.e., ICC = 0.87) in that study, perhaps due to use of the 12-item version 1.0 short form, rather than the 10- and 20-item version 2.0 PROMIS-PF short forms used in the current study. To our knowledge, the LEFS has not been examined for evidence of reliability in LLO users, but has been shown to have high reliability (i.e., 0.85–0.99) across a wide range of clinical populations,[24] many of whom might require use of a LLO to address the mobility limitations caused by their health condition.

The SEM and SDC values derived in the current study provide important context for interpretation of OPRO-M scores. With an SDC90 of 6.5 points on the T-score metric, administrators can be 90% confident that score changes exceeding this threshold represent true change rather than measurement error. This relatively small SDC value, compared to the 12.7 points for LEFS, suggests that OPRO-M short forms may be more sensitive to detecting meaningful changes in mobility status over time. However, additional research will be needed to assess OPRO-M’s sensitivity to change relative to these other self-report instruments.

The strong psychometric properties demonstrated by both OPRO-M 12- and 20- item short forms in this study provide clinicians and researchers with greater confidence in using them to assess mobility in LLO users. The OPRO-M 12-item short form is recommended in most situations, including where orthotic mobility is a primary outcome (e.g., comparative effectiveness studies) or when monitoring individual patients (e.g., measuring mobility after delivery of a LLO). The OPRO-M 20-item short form measures with higher precision at the extreme ends of the scale and may be more suitable when measuring individuals with very low or very high mobility. The evidence of validity and reliability supports OPRO-M’s use for various clinical and research applications, including initial assessment, treatment planning, outcome evaluation, and comparative effectiveness research. OPRO-M offers several benefits to administration in routine clinical practice. Its development specifically for LLO users ensures that the content is relevant to this clinical population [7,8,49], potentially making it more acceptable to patients and more informative to clinicians than generic self-report instruments. The availability of short forms reduces administrative burden while maintaining measurement precision, facilitating integration into busy clinical workflows. Additionally, the T-score metric enables clear interpretation relative to a reference population of orthosis users, aiding in meaningful communication of results to patients and other healthcare providers.

While PROMIS-PF demonstrated similarly strong psychometric properties in this study, OPRO-M’s population-specific focus provides advantages in item relevance and score interpretation for LLO users. PROMIS-PF is calibrated to the general U.S. population, which may limit its sensitivity to detect mobility changes specific to orthosis users. For example, a prior study found that AFO users reported a small range of PROMIS-PF T-scores and had a much lower mean score than the normative sample (i.e., 30.8 vs 50.0) [50]. OPRO-M, calibrated specifically to orthosis users, provides a more targeted measurement tool that may better reflect clinically meaningful changes in this population. The ability of OPRO-M short forms to differentiate between mobility groups, particularly in the middle-to-upper ranges of mobility, reinforces its utility for tracking progress as patients advance from limited community ambulation to higher levels of function. However, the findings also indicate that clinicians may benefit from using a combination of self-report and performance-based instruments, particularly when assessing individuals with lower mobility. The TUG’s ability to distinguish between lower mobility groups highlights the value of a comprehensive assessment approach that incorporates both patient-reported and performance-based instruments.[3]

Several limitations should be considered when interpreting the results of this study. The use of convenience sampling resulted in uneven representation of certain health conditions and orthosis types in our sample. This may limit the generalizability of findings to all LLO users, particularly those with less common conditions or using less common orthosis designs. Future validation studies with targeted recruitment of underrepresented groups would strengthen the evidence base for OPRO-M’s use across the full spectrum of LLO users. The mobility classification system used in this study relied on clinician judgment rather than standardized criteria, which may have introduced subjectivity in group assignments. Future studies might alternatively employ diagnosis-specific mobility classifications (e.g., American Spinal Injury Association Impairment Scale [51]) to further validate OPRO-M’s discriminative ability. Our study evaluated test-retest reliability over a relatively short period (7–16 days), which is appropriate for assessing measurement stability but does not address the instrument’s responsiveness to change. Additional research examining OPRO-M’s sensitivity to detect clinically meaningful changes following provision of an intervention would provide valuable information about its utility for longitudinal monitoring. Finally, while our sample size was adequate based on power calculations and COSMIN recommendations, larger samples would enable more detailed subgroup analyses to evaluate OPRO-M’s performance across specific health conditions, orthosis types, and demographic characteristics.

## Conclusions

The OPRO-M short forms demonstrate strong evidence of validity and reliability for measuring mobility in LLO users. Their performance is comparable to or better than existing self-report instruments, with the added benefits of a population-specific focus and inherent reference to large, national population of LLO users. The relatively low measurement error associated with both OPRO-M short forms make them suitable for use in both clinical and research settings. OPRO-M short forms are available at https://opro-m.org. Future research should focus on evaluating OPRO-M’s responsiveness to change and developing specialized tools for assessing individuals with lower mobility levels. With strong psychometric properties and a targeted focus on LLO users, OPRO-M represents an important advancement in outcomes assessment for the lower limb orthotic patient population.

## Supporting information

S1 File Administration protocols for performance-based instruments. (PDF)

S2 File Dataset used to perform analyses. (XLSX)

S1 Fig Correlations among the comparison self-report instrument scores. Lower Extremity Functional Scale (LEFS) total scores were strongly correlated with PROMIS Physical Function (PROMIS-PF) 20-item Short Form T-scores (ρ = 0.90). PROMIS-PF 20-item Short Form T-scores were strongly correlated with Orthotics and Prosthetics Users Survey – Lower Extremity Functional Status (OPUS-LEFS) Rasch measures (ρ = 0.92). OPUS-LEFS Rasch measures were strongly correlated with LEFS total scores (ρ = 0.90).
(TIF)

S2 Fig Correlations between comparison self-report instrument scores and performance-based instrument scores. PROMIS Physical Function (PROMIS-PF) 20- and 10-item Short Form T-scores scores were moderately correlated with Timed Up and Go (TUG) times (both ρ = −0.67) and 10-meter Walk Test (10mWT) speed (both ρ = 0.67), and strongly correlated with Two-Minute Walk Test (2MWT) distances (ρ = 0.73 and 0.74, respectively). Lower Extremity Functional Scale (LEFS) total scores were moderately correlated with TUG times (ρ = −0.67), and 10mWT speed (ρ = 0.68), and 2MWT distances (ρ = 0.75). Orthotics and Prosthetics Users Survey – Lower Extremity Functional Status (OPUS-LEFS) Rasch measures moderately correlated with TUG times (ρ = −0.68), and strongly correlated with 10mWT speed (ρ = 0.71) and 2MWT distances (ρ = 0.75).
(TIF)
