# Supplementary material for: Evaluating the construct validity and test-retest reliability of the Orthotic Patient-Reported Outcomes–Mobility (OPRO-M) short forms in lower limb orthosis users
Source: PLoS One. 2025 Aug 19;20(8):e0330334. doi: 10.1371/journal.pone.0330334 (PMC12364370; doi:10.1371/journal.pone.0330334)
Supplement: S1 File — (PDF) [file pone.0330334.s001.pdf]

S1 File. Administration protocols for performance-based instruments.

Timed Up and Go Test (TUG)

Description: The Timed Up and Go (TUG) test is a performance-based outcome measure developed to assess an individual's ability to rise from a chair, walk 3m, turn around, return to the chair, and be seated.

Equipment: Stopwatch; standard height chair with straight back (43cm height, 47.5cm seat depth); cone.

Setup: Place the cone 3m in front of the chair, as shown below. For safety, place the chair against a wall or secure the chair as the person stands up and sits down.

Administration:

- Have the participant start the test seated with their back against the chair and their arms resting on the armrests.
- Inform them that, on the word "go" they are to get up and walk around the cone, return to the chair, and sit down again. Remind them they are to walk at a comfortable speed.
- Demonstrate the test. Have the participant practice once before being timed in order to become familiar with the test.
- Begin timing when you say "Go" and stop when the participant's buttocks touch the chair.
- Administer the test two times. Record both test times.

Verbal Instructions: "Begin the test sitting with your back against the back of the chair and your arm resting on the armrests. When I say 'go,' please stand up and walk around the cone, walk back to the chair, and sit down again. Please walk at your normal, comfortable pace. Remember the goal of this test is to walk at a comfortable speed. I will give you a countdown. Begin when I say 'go.' Are you ready? 3...2...1...go."

10 Meter Walk Test (10mWT)

Description: The 10 Meter Walk Test (10mWT) is a performance-based outcome measure developed to assess an individual's comfortable walking speed.

Equipment: Stopwatch, tape, tape measure (metric), four cones.

Setup: Prepare a 14-meter walkway in a hallway or other unobstructed area. Place tape lines at 0, 2, 12, and 14 meters. Mark the lines at 0 and 14 meters with cones, as shown below.

Administration:

- Have the participant start in a standing position on the 0-meter line.
- Inform them that, on the word "go," they are to walk at a comfortable speed until you say "stop."
- Demonstrate the test. Have the participant practice once before being timed in order to become familiar with the test.
- Begin timing when the participant crosses the 2-meter line. Stop timing when the participant crosses the 12-meter line. Inform the participant to stop when they cross the 14-meter line.
- Administer the test two times. Record both test times.

Verbal Instructions: "Begin the test sitting with your back against the back of the chair and your arm resting on the When I say 'go,' please walk at your normal, comfortable pace until I say stop. Remember the goal of this test is to walk at a comfortable speed. I will give you a countdown. Begin when I say 'go.' Are you ready? 3...2...1...go."

2 Minute Walk Test (2MWT)

Description: The 2 Minute Walk Test (2MWT) is a performance-based outcome measure developed to assess an individual's functional mobility and walking speed.

Equipment: Stopwatch, 2 cones, lap counter, tape measure or measuring wheel (metric).

Setup: Set two cones 10m apart in a clear, open area (e.g., a hallway with minimal foot traffic). Designate the area with signs to minimize interruption if needed.

Administration:

- Have the participant start in a standing position to one side of one cone.
- Inform them that, when they are ready, they are to walk around the outside of the cones (in their preferred direction) at a fast speed until you say "stop."
- Demonstrate the test. Do not administer a practice trial.
- Begin timing when the participant starts walking. Use the counter to note each time the participant passes a cone. Provide encouragement to the participant as noted below. Note the spot where the participant was when 2 minutes expired with the beanbag, and then ask them to stop.
- Administer the test one time. Record the distance.

Verbal Instructions: "The goal of this test is to walk as far as possible for 2 minutes. You will walk around the two cones [point them out]. You will probably get out of breath or become exhausted. You are permitted to slow down, to stop, and to rest as necessary. Resume walking as soon as you are able. You will be walking around the cones. You should turn quickly around each cone. Now I'm going to show you. Please watch the way I turn quickly around the cone. Are you ready to do that? Remember that the object is to walk AS FAR AS POSSIBLE for 2 minutes, but don't run or jog. Start now or whenever you are ready."

Encouragement: Provide encouragement every minute. See standard phrases below:

- After minute 1: "You are doing well. You have 1 minute to go."
- 15 seconds from completion: "In a moment, I am going to tell you to stop. When I do, just stop where you are and I will come to you."

Note: Do not use other words of encouragement or body language to speed up. If the patient stops during the test, say this: "You can lean against the wall if you would like; then continue walking when you are able." Do not stop the timer. If the patient stops before the 2 minutes are up and does not want to continue (or you think they should not continue), bring a chair for the participant to sit on, note the distance and the reason for stopping prematurely.
