# Supplementary figures and images for: Evaluating the construct validity and test-retest reliability of the Orthotic Patient-Reported Outcomes–Mobility (OPRO-M) short forms in lower limb orthosis users
Source: PLoS One. 2025 Aug 19;20(8):e0330334. doi: 10.1371/journal.pone.0330334 (PMC12364370; doi:10.1371/journal.pone.0330334)

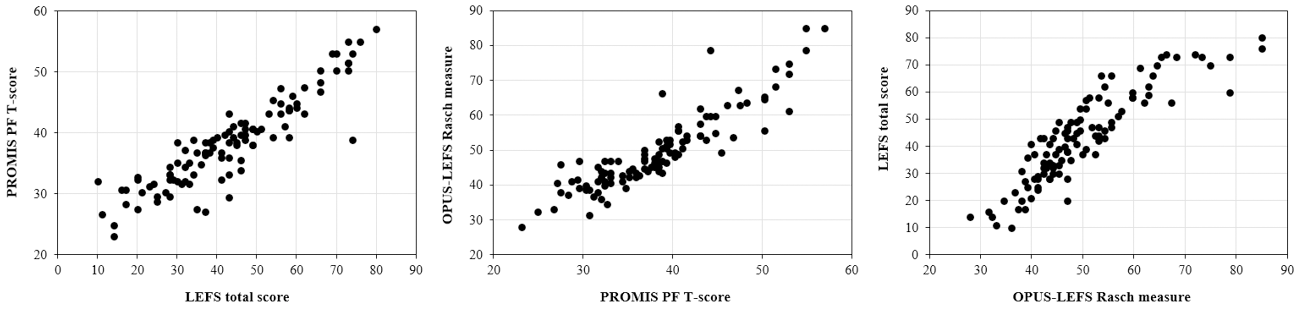

Supplement: S1 Fig — Lower Extremity Functional Scale (LEFS) total scores were strongly correlated with PROMIS Physical Function (PROMIS-PF) 20-item Short Form T-scores (ρ = 0.90). PROMIS-PF 20-item Short Form T-scores were strongly correlated with Orthotics and Prosthetics Users Survey – Lower Extremity Functional Status (OPUS-LEFS) Rasch measures (ρ = 0.92). OPUS-LEFS Rasch measures were strongly correlated with LEFS total scores (ρ = 0.90). (TIF) [file pone.0330334.s003.tif]

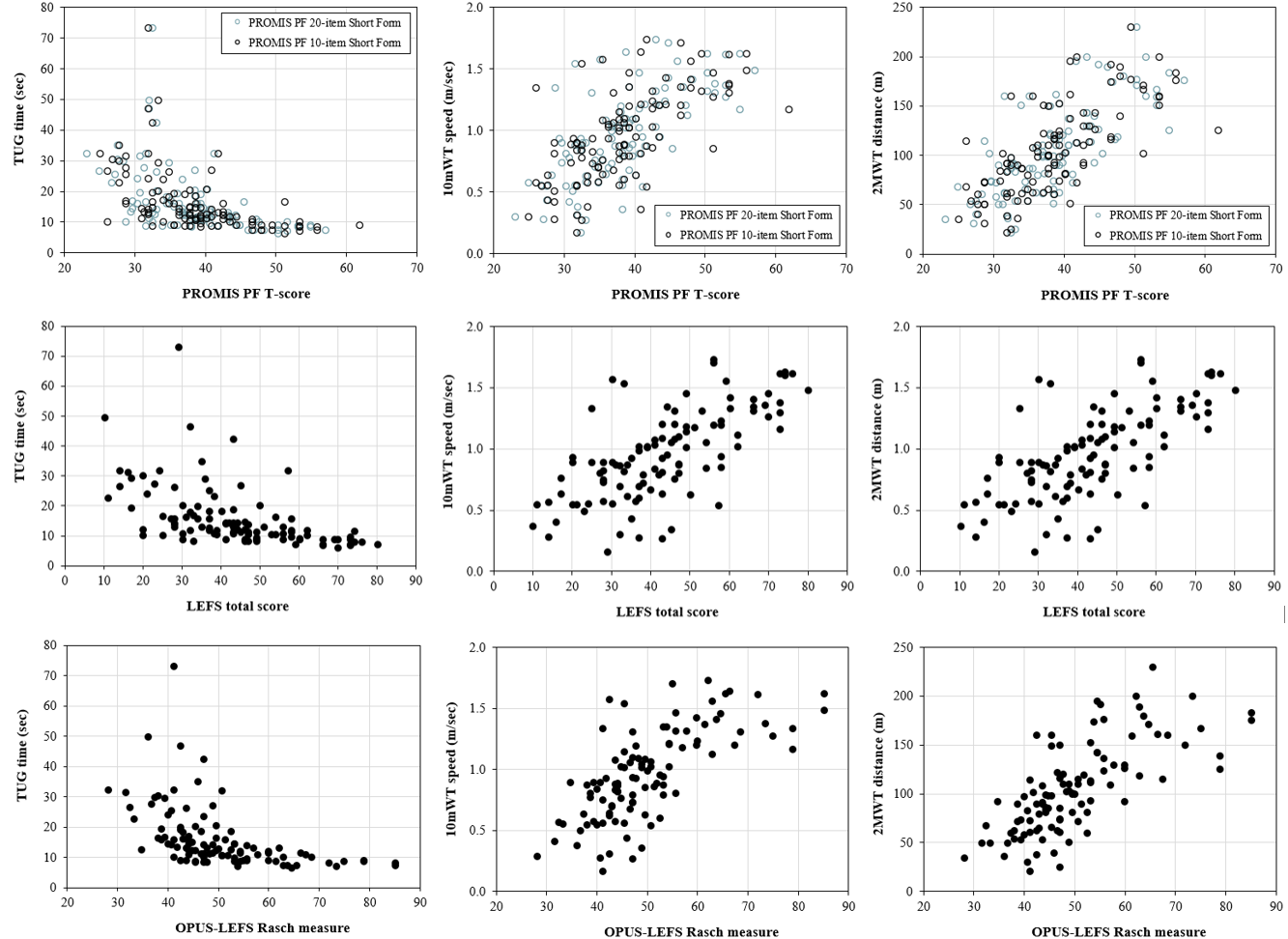

Supplement: S2 Fig — PROMIS Physical Function (PROMIS-PF) 20- and 10-item Short Form T-scores scores were moderately correlated with Timed Up and Go (TUG) times (both ρ = −0.67) and 10-meter Walk Test (10mWT) speed (both ρ = 0.67), and strongly correlated with Two-Minute Walk Test (2MWT) distances (ρ = 0.73 and 0.74, respectively). Lower Extremity Functional Scale (LEFS) total scores were moderately correlated with TUG times (ρ = −0.67), and 10mWT speed (ρ = 0.68), and 2MWT distances (ρ = 0.75). Orthotics and Prosthetics Users Survey – Lower Extremity Functional Status (OPUS-LEFS) Rasch measures moderately correlated with TUG times (ρ = −0.68), and strongly correlated with 10mWT speed (ρ = 0.71) and 2MWT distances (ρ = 0.75). (TIF) [file pone.0330334.s004.tif]
